# The influence of laser-induced alignment on Z-scan properties of 2D carbon nanomaterials suspension dependent on polarization

**DOI:** 10.1038/s41598-022-14577-0

**Published:** 2022-06-16

**Authors:** Qiuhui Zhang, Xinghui Wu, Jinghua Han

**Affiliations:** 1 Department of Electrical Information Engineering, Henan University of Engineering, Xinzheng, 451191 Henan China; 2 College of Electronics and Information Engineering, Sichuan University, Chengdu, 610064 China

**Keywords:** Nanoscience and technology, Optics and photonics

## Abstract

The Z-scan technique uses a single beam that can be used for observing the nonlinear or optical limiting properties of materials. For the first time, the Z-scan properties dependent on the polarization of 2D carbon nanomaterial suspension were experimentally investigated using optical Z-scan technology. The Z-scan curves of graphene and graphene oxide (GO) in *N*-methyl-2-pyrrolidinone suspensions exhibited strong polarization-dependent characteristics. In paper, a reverse saturated absorption (RSA) dip surrounded the lens focus when the horizontal polarized beam was focused in the suspension, and two saturated absorption (SA) peaks appeared adjacent to the dip. However, for the vertical polarized beam, only one RSA dip surrounded the lens focus, and the threshold was higher than the SA for a horizontally polarized beam. The transmission of RSA for the GO suspension was evidently lower than that of the graphene suspension. The polarization-dependent characteristic can be ascribed to the laser-induced alignment in case the suspension is moved in or out of the beam focal point. Furthermore, the polarization-dependent 2D carbon nanomaterial suspension can be applied in several practical purposes such as 2D material-based optical and opto-fludic devices.

## Introduction

The Z-scan technique is used to measure optical nonlinearities of materials, including nonlinear absorption and refraction properties^1,2^. Carbon nanomaterials have garnered considerable interest owing to their unique properties and fascinating structure for utilizing the nonlinear properties of carbonaceous matter at the nanoscale, using an open-aperture^3–7^ or closed-aperture Z-scan technique^8,9^, especially graphene and its derivatives^6,10–12^. Optical limiting is an important property of materials and can be typically obtained using the open-aperture Z-scan technique, which can control the light intensity in a predetermined and predictable manner in addition to prevent laser damage. Several researchers have studied the optical limiting phenomenon^13–16^. Recently, the Z-scan properties of nanomaterial suspensions have attracted scholarly interest^13,14,17–22^. In particular, a liquid environment can provide nanostructures with more degrees of freedom in both rotational and translational motions as well as enable us to study the collective properties of macroscopically ordered assembly of nanomaterials—a condition that remains unrealized in their solid state^23,24^. Moreover, 1D or 2D nanomaterials are intrinsically anisotropic in shape, electronic, and optical properties. Therefore, their liquid suspensions can exhibit isotropic or anisotropic properties macroscopically depending on the microscopic alignment of suspended nanostructures^11,23,25–27^. An important consequence of this dynamic alignment involves the modulation of the incident laser: the aligned nanostructures will increase or decrease the optical transmission depending on the laser polarization. However, the dependence of the Z-scan properties depend on polarization is still unclarified through research, because it should not matter for either saturated absorption (SA) or reverse saturated absorption (RSA), especially for graphene and graphene oxide (GO) suspensions. Graphene has been reported to exhibit polarization-dependent optical absorption in the visible spectral range^28,29^, ultrafast carrier dynamics^30^, and double-resonant Raman^31–33^. Furthermore, black phosphorus exhibits anomalous polarization dependence of Raman scattering and crystallographic orientation^34^.

With pioneering novelty, this study reports the influence of polarization on the Z-scan properties of a 2D carbon nanomaterial suspension under Z-scan technology. In particular, the graphene and GO in the N-methyl-2-pyrrolidinone (NMP) and deionized (DI) water suspensions displayed polarization dependence, and the Z-scan results slightly varied for the graphene and GO suspensions.

## Methods

Few-layer graphene flakes with a lateral dimension of a few micrometers are synthesized via intercalation and exfoliation of natural graphite. Single-layer graphene oxide (GO) flakes with sizes in the range of 2 ~ 10 μm are prepared by a modified Hummers’ method^35^. As-obtained graphene and graphene flakes are dispersed in DI water or N-methyl-2-pyrrolidone (NMP) by sonication and form stable suspensions with a concentration of 0.005–0.01% in weight percentage (wt%), corresponds to 0.002–0.004% in volume fraction (vol%).

## Results and discussion

**Figure 1:**
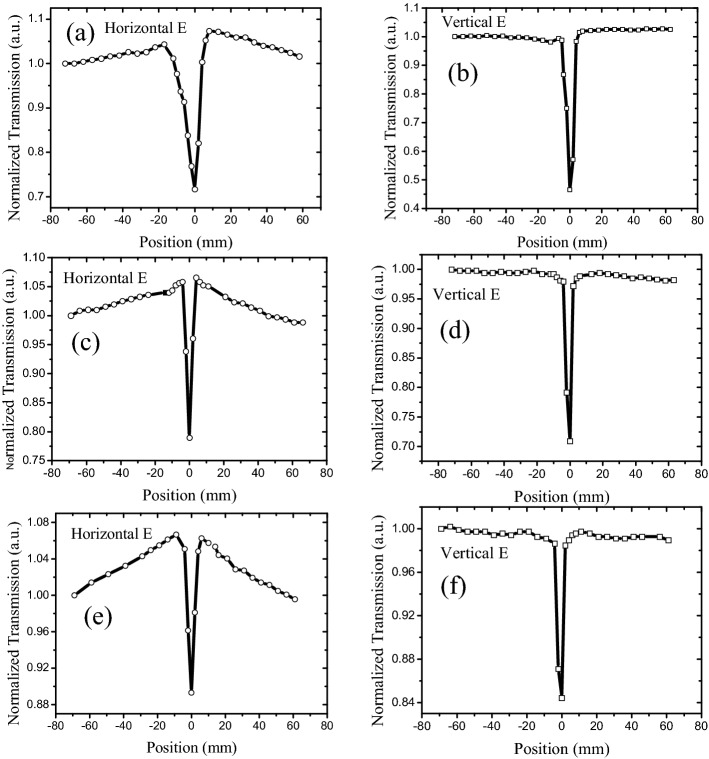
Z-scan results of graphene suspended in NMP: **(a,b)** 527-nm laser with 100 mW power; transmission of graphene suspension is 10%; cuvette is 10 mm wide; **(c,d)** 527-nm laser with 50 mW power; transmission of graphene suspension is 10%; cuvette is 5 mm wide; **(e,f)** 527-nm laser with 50 mW power; transmission of graphene suspension is 60%; cuvette is 10 mm wide.

The Z-scan properties of the carbon nanostructure suspension were measured using a traditional open-aperture Z-scan system, wherein the scan laser beam (527 nm wavelength, 150 ns pulse width, 1 kHz repetition rate) was a polarized beam focused with a lens of 10 cm focal length. The Z-scan results of graphene in the NMP suspension under horizontally and vertically polarized beams are illustrated in Fig. 1.

As depicted in Fig. 1, the Z-scan properties observed under a horizontally polarized beam varied from those detected using a vertically polarized beam. In addition to RSA-like dip surrounding the lens focus when the horizontally polarized beam is focused in suspension, two SA-like peaks appeared adjacent to the dip. However, for the vertical polarized beam, only one RSA-like dip surrounded the lens focus. In contrast, two SA-like peaks were distributed on both sides of the focus (RSA dip) for the horizontally polarized beam, implying that the SA-like peaks were situated far away from the focus, and the threshold was lower than that of the RSA-like dip. Simultaneously, the lowest normalized transmission of the horizontally polarized beam (Fig. 1a) was higher than that of the vertically polarized beam (Fig. 1b), which indicated that the RSA-like dip peculiarity of the vertically polarized beam was stronger than that of the horizontally polarized beam. Regardless of the SA peak and RSA, the dip increased with the laser energy (comparing Fig. 1a,c) and decreased with the graphene concentration (comparing Fig. 1c,e).

**Figure 2:**
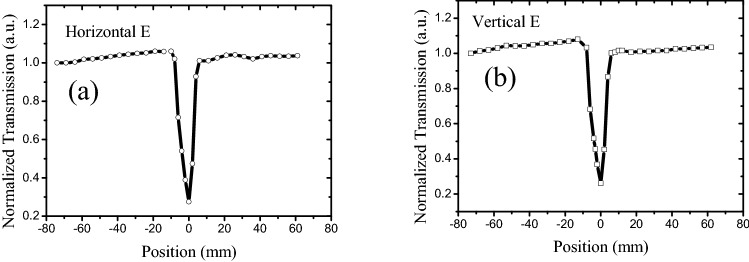
Z-scan results of graphene suspended in DI water. 527-nm laser with 100 mW power; transmission of grapheme suspension is 10%; cuvette is 10 mm wide.

The influence of the solvent on the polarized Z-scan properties of the graphene suspension was investigated by using DI water as the solvent to obtain a graphene–DI water suspension, whose viscosity coefficient varied from that of the NMP^36,37^; the results are displayed in Fig. 2. Upon using a horizontally polarized beam, the SA peaks almost disappeared for the graphene–DI water suspension (Fig. 1). However, the SA characteristics were affected by the solution because the RSA dip remained.

**Figure 3:**
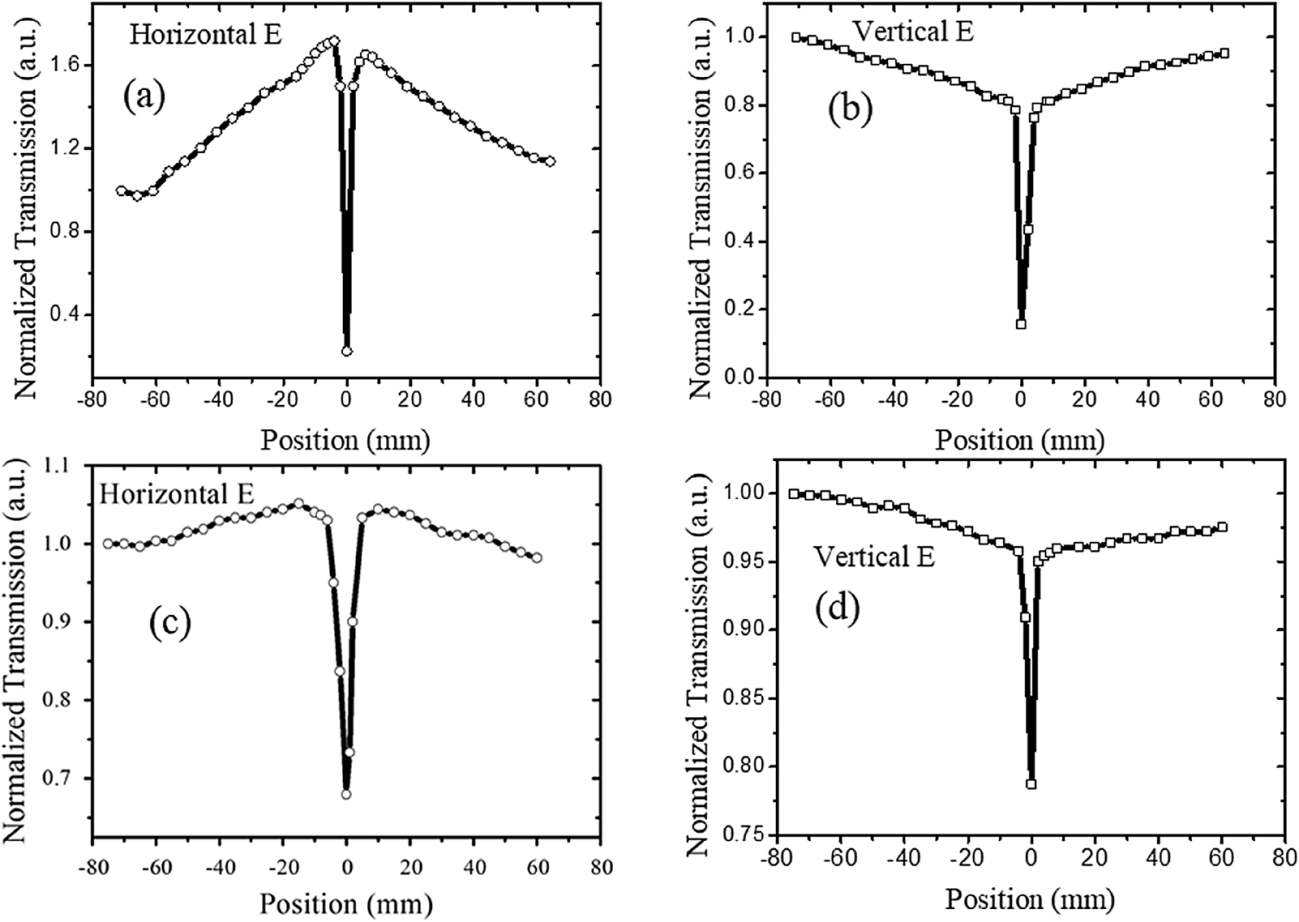
Z-scan results of GO in NMP. **(a,b)** 527-nm laser with 50 mW power; transmission of graphene suspension is 10%; cuvette is 5 mm wide; **(c,d)** 527-nm laser with 50 mW power; transmission of graphene suspension is 60%; cuvette is 10 mm wide.

**Figure 4:**
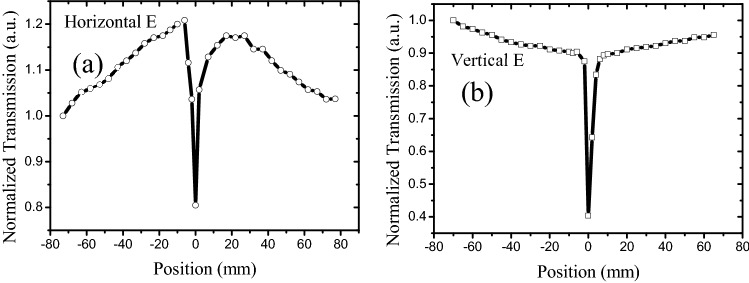
Z-scan results of GO in DI water. 527-nm laser with 50 mW power; transmission of graphene suspension is 10%; cuvette is 5 mm wide.

The Z-scan results of GO in the NMP and DI water suspensions are illustrated in Figs. 3 and 4, respectively. Regardless of GO in the NMP or DI water suspension, SA- and RSA-like curves were observed for the horizontally polarized beams, but the RSA was observed for vertical polarization at the same incident laser power (Figs. 3 and 4). Moreover, the SA transmission of the GO–NMP suspension (Fig. 3a) was evidently higher than that of the GO–DI water suspension (Fig. 4a). Subsequently, the SA peaks disappeared as the transmission of the GO–NMP suspension increased from 10 to 60% (Fig. 3c), signifying that the concentration of the GO–NMP suspension could affect the Z-scan properties under a polarized beam. In summary, the polarized properties of the scan laser and the concentration of the suspension could affect the Z-scan properties of the GO–NMP suspension, but the influence of the solvent almost disappeared.

**Figure 5:**
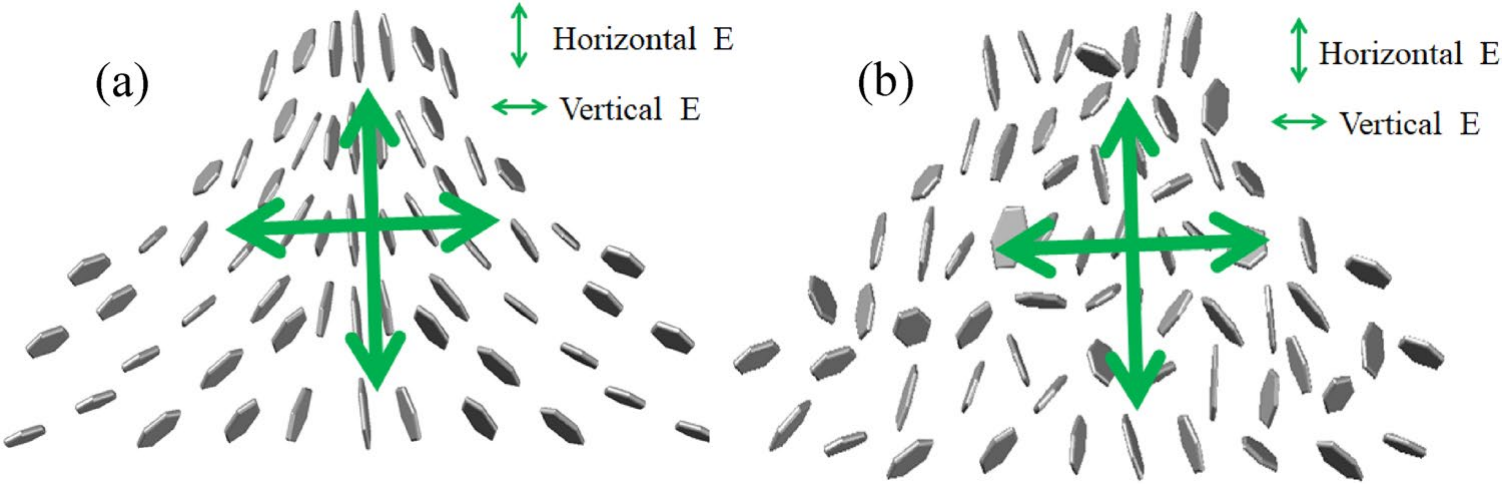
Schematic of transmission of polarization laser goes through suspension. **(a)** Orientations of graphene flakes in case suspension out of focus; **(b)** misalignment of graphene flakes when focused laser passes through suspension.

In case a high-intensity laser was incident on the suspension, the suspended nanostructures absorbed the incident laser energy and converted it into heat. Subsequently, the heat was transferred to the surrounding liquid, creating a local temperature gradient and natural convection. As the simulation results of flow velocity distribution by computational fluid dynamics (CFD) in our paper^38^, there is barely any flow when intensity laser irradiate 0.4 s, but increases considerably at 4 s. In general, a flake will experience imbalanced shear forces on its head and tail ends, which produces a net torque causing rotation of the flake^39^. In addition, laser-induced dynamic alignment and optical anisotropy of nanomaterials further result in polarization-dependent modulation of laser transmission^38^. However, the degree of laser-induced alignment varied in case the suspension was moved in or out of the focal point of the beam, and it was related to the laser intensity. Moreover, low laser intensity was observed with weak laser-induced temperature gradient and subsequent fluidic flows in case the suspension was out of focus. In comparison, a much stronger flow and subsequent alignment could be observed if the focused beam was in the vicinity of the suspension, and the flakes were vertically aligned, as depicted in Fig. 5a. Therefore, an enhanced optical transmission was produced under the horizontally polarized beam in the Z-scan experiment^23,24^, and two SA-like peaks appeared under horizontally polarized cases, as depicted in Figs. 1, 3, and 4. However, the vertically aligned nanomaterials blocked and scattered the incident laser (vertically polarized laser), even if the 2D carbon nanomaterials were aligned, as depicted in Fig. 5a. Therefore, the SA-like peaks did not appear in the vertically polarized case. In a word, the SA-like transmission is the result of modulation of laser induced dynamic alignment and optical anisotropy of nanomaterials, which is not the third-order nonlinear. An important criterion to determine whether the observed nonlinear-like transmission induced by third-order susceptibility χ^(3)^ is the laser pulse width, and only sub-picosecond or short than sub-picosecond with high peak intensity can meet the timescale of electronic transitions^40–42^. But for most nanosecond laser pulses, they are too low to induce a significant nonlinear optical effect despite the observation of obvious nonlinear-like Z-scan transmission. In Fig. 2, the SA peaks did not appear for the graphene in DI water suspension under a horizontally polarized beam, which reflected that graphene cannot be appropriately aligned in the DI water as compared to the NMP solution (Fig. 1). This signified that the dispersed liquid could affect the alignment of the graphene.

However, when the focused beam was transmitted through the suspension, the highest laser intensity, highest laser-induced temperature gradient, and fastest fluidic flow caused by convection were observed at this spot. Thus, the alignment was strongly disturbed and misaligned, as depicted in Fig. 5b. In case the alignment was disintegrated, the 2D carbon nanomaterials blocked and scattered the incident laser. Simultaneously, as mentioned in our previous work^43^, the carbon nanomaterials absorbed the energy of the incident laser and transferred to surrounding liquid, the Raman experiment result showed that the temperature rise is only 180 °C, which is just slightly higher than the boiling temperature of water, resulting in vapor and microbuubles generation. At the same time, the CCD and ultrasound results further verified the existence of photothermal bubbles near the focus. Moreover, the photothermal microbubble strongly scattered the incident laser, which induced a normalized transmission turn-down after exhibiting the SA-like peak. The focused beam exhibited a small beam radius with an increasing number of photothermal microbubbles in the vicinity of the focus, and the scatter intensified when the focused laser passed through the suspension. Therefore, the scattering induced by the photothermal microbubbles were independent of the polarization, and both the horizontally and vertically polarized cases displayed an RSA-like dip, as depicted in Figs. 1, 2, 3 and 4.

**Figure 6:**
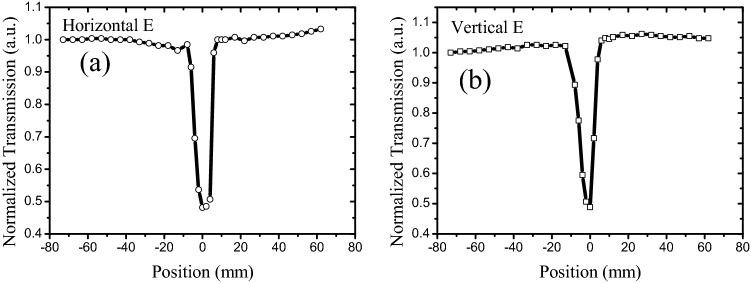
Z-scan results of carbon particles in DI water. 527-nm laser with 50 mW power; transmission of carbon particles suspension was 15%; cuvette was 10 mm wide.

To further verify the polarization-dependent transmission induced by the laser-induced alignment of nanomaterials in suspension, the carbon particle suspension was selected to derive various results. Consequently, the isotropic particles and Z-scan properties of the carbon particles suspended in DI water are represented in Fig. 6. The obtained results varied from those obtained for graphene and GO suspensions; the SA peak disappeared for the horizontally polarized beam, and only the RSA dip and RSA-like curve remained for the vertically polarized beam. Overall, the Z-scan curve for the horizontally polarized beam was almost identical to that of the vertical polarized beam, which implied that the Z-scan properties of the carbon particle–DI water suspension were independent of the polarized properties of the scan beam. As the carbon particles were isotropic, its isotropic properties allowed a uniform transmission of the light in all directions, even if the carbon particles were aligned by the incident laser. Therefore, the SA-like peaks were not observed for the carbon particles in DI water suspension under a horizontally polarized beam, as depicted in Fig. 6a.

## Conclusion

In summary, open-aperture Z-scan results revealed that graphene and GO flakes exhibited SA- and RSA-like curves under horizontally polarized beams, whereas only the RSA-like curves were observed for vertical polarization under the same conditions. Moreover, the RSA-like dip of the GO suspension was narrower than that of the graphene suspension. In particular, the RSA thresholds of the graphene and GO suspensions were lower for the horizontal beam than those for the vertical beam. These results signified that the Z-scan characteristics of graphene and GO suspensions depend on the polarization of the incident laser, and the suspension particles were anisotropic, which could be ascribed to the laser-induced dynamic alignment of graphene or GO flakes. However, the Z-scan properties of the isotropic materials such as carbon particles are independent of the polarization of the incident laser. Simultaneously, the solvent of the graphene suspension and concentration of the GO suspension influenced the SA-like peaks under polarized laser. Furthermore, the SA-like peaks disappeared in both the graphene–DI water suspension and the high-concentration GO suspension, and only the RSA-like dip remained. Thus, the anisotropic optical responses of the liquid suspensions provided innovative routes for studying the fundamental light–matter interaction and the Z-scan curve principle, which is essential for novel 2D material-based optical and optofluidic devices.
